# The perception of air pollution and its health risk: a scoping review of measures and methods

**DOI:** 10.1080/16549716.2024.2370100

**Published:** 2024-06-28

**Authors:** Zeinab Bahrami, Satomi Sato, Zhesi Yang, Monali Maiti, Paoin Kanawat, Tomohiro Umemura, Kazunari Onishi, Hiroaki Terasaki, Tomoki Nakayama, Yutaka Matsumi, Kayo Ueda

**Affiliations:** a Research Institute for Humanity and Nature, Kyoto, Japan; b Graduate School of Engineering, Kyoto University, Kyoto, Japan; c Graduate School of Public Health, St Lukes International University, Chuo, Japan; d Department of Chemistry and Forensic Science, R.B.V.R.R. Women’s College, Hyderabad, India; e Department of Health and Psychosocial Medicine, Aichi Medical University School of Medicine, Nagakute, Japan; f Faculty of Engineering, University of Fukui, Fukui, Japan; g Faculty of Environmental Science, Nagasaki University, Nagasaki, Japan; h Institute for Space-Earth Environmental Research, Nagoya University, Nagoya, Japan; i Graduate School of Medicine, Hokkaido University, Sapporo, Japan

**Keywords:** Air pollution, air quality, health risk, health risk perception, standardised scales, awareness

## Abstract

**Background:**

Although there is increasing awareness of the health risks of air pollution as a global issue, few studies have focused on the methods for assessing individuals’ perceptions of these risks. This scoping review aimed to identify previous research evaluating individuals’ perceptions of air pollution and its health effects, and to explore the measurement of perceptions, as a key resource for health behaviour.

**Methods:**

The review followed the methodological framework proposed by Arksey and O’Malley. PubMed and Web of Science were searched. After initial and full-text screening, we further selected studies with standardised scales that had previously been tested for reliability and validity in assessing awareness and perceptions.

**Results:**

After full-text screening, 95 studies were identified. ‘Perception/awareness of air quality’ was often measured, as well as ‘Perception of health risk.’ Only nine studies (9.5%) used validated scaled questionnaires. There was considerable variation in the scales used to measure the multiple dimensions of risk perception for air pollution.

**Conclusion:**

Few studies used structured scales to quantify individuals’ perceptions, limiting comparisons among studies. Standardised methods for measuring health risk perception are needed.

## Paper Context

- **Main findings:** Among 95 studies assessing health risk perception of air pollution, only nine studies used standardised scales.
- **Added knowledge:** There was considerable variation in the scales measuring the multiple dimensions of risk perception for air pollution, which makes comparison among the studies difficult.
- **Global health impact for policy and action:** This review highlights the need for the development of globally standardised scale to measure the health risk perception of air pollution.

## Background

Risk perception refers to individuals’ assessments and judgements towards potential hazards [1], which involves subjective cognitive processes and individual-level factors, such as a person’s experiences, feelings, knowledge, and beliefs, as well as scientific judgement. Risk perception is a key component in the formation of attitudes towards policies, as well as individuals’ behaviours. A number of studies have focused on the effects of risk perception on specific health-protective actions, and it is widely accepted that risk perception is a determinant of changes in health behaviour [2]. For example, previous studies have suggested an association between risk perception regarding infectious diseases and preventive behaviours, such as vaccination [3], travel avoidance [4], and distancing behaviours in response to infectious diseases [5].

Other studies have examined risk perception of environmental hazards, such as flooding [6], climate change [7], and radiation [8]. It has been reported that risk perception of a specific environmental hazard can affect individuals’ behaviours regarding avoidance of the associated health consequences [9,10] and modify their attitudes towards specific policies to reduce the population risk [11,12]. There are arguments both for and against the use of risk perceptions in policy [13–15].

Regarding environmental hazards, in recent years there have been an increasing number of studies focusing on the risk perception of air pollution [16]. Individuals’ behaviours and intentions regarding the prevention of exposure to air pollution depend on how they perceive the health risks associated with air pollutants. One previous study showed that the perceived severity of air pollution-related health risks was associated with behavioural changes, including changing the duration and intensity of outdoor activity and the usage of air purifiers [17]. Another survey conducted in Singapore examined respondents’ risk perception regarding haze pollution, the primary source of which was forest fires in Southeast Asia; the results suggested that attention to media, interpersonal discussions, and knowledge played important roles in shaping individuals’ risk perception regarding haze, and changed people’s attitudes towards taking preventive measures [18]. These results provide important evidence supporting health policies to raise public awareness about air pollution and encouraging preventive behaviours.

The heterogeneity in the results in existing studies of risk perception of air pollution and its health risks may originate from differences in the methods used to characterise individuals’ risk perception, given the nature of subjective assessment and judgement. Additionally, risk perception has various conceptual dimensions [16]. Most previous studies have focused on the specific dimensions of risk perception, which makes comparisons among studies difficult. Exploring the methodologies used for assessing risk perception of air pollution in previous studies may be useful for facilitating our understanding of the processes of behavioural change in the context of air pollution and health. Therefore, the current study aimed to identify literature evaluating people’s perceptions of the health risks of air pollution and to explore the methodologies used in these previous studies. Specifically, we focused on the scales sin the studies and addressed the following research questions.

1. What are the available scales for measuring the perception of air pollution and its health risks?
2. Which dimensions of perception do the scales measure?

We also explored the availability of scales targeting children because scales designed for adults may not always be applicable to children without modification.

## Methods

We followed a five-stage methodological framework that was initially outlined by Arksey and O’Malley [19] and further advanced by Levac [20].

### Literature search

We searched PubMed and Web of Science using the following keywords: air pollution (‘air pollution’, ‘air quality’), perception (‘perception’, ‘perceived health risk’, ‘perceived risk’) and awareness (‘awareness’). The literature search was restricted to articles published between 1 January 1970 and 15 March 2022. Non-English language articles and grey literature were not included.

### Study selection

We included original studies evaluating the perception of health risk from outdoor air pollution. Articles evaluating awareness, concerns, attitude and behaviour were also included as far as these concepts were related to outdoor air pollution or air quality. We excluded (1) studies related to indoor or household air pollution, tobacco, cigarettes, fumes and dust from occupational exposure, pesticides, radon, and asbestos, (2) reviews, commentaries, editorials, and letters, (3) experimental studies (as well as choice experiment), interventions, and (4) studies based on analyses of data from social networking sites (i.e. no direct or registered participants).

Four reviewers separately screened titles and abstracts. The full text of the selected article was reviewed by four authors. Disagreements regarding study selection were resolved by discussion between the reviewers.

### Data extraction, processing, and reporting

For each article included in the analysis, we extracted the following data: bibliographic information, study objectives, study area and time period, participants’ demographic information, sample size, and the methods of the survey. We classified the region according to ‘Our World in Data.’ We extracted the key dimensions of perceptions related to health risks from air pollution: ‘perception of health risk,’ which was considered to reflect a person’s beliefs, judgements, and feelings [14]; ‘perception/awareness of air quality,’ which was considered to reflect a person’s tendency to think about or be informed about the level of air quality [21], ‘attitude,’ which was considered to refer to the degree of a person’s favourable/unfavourable evaluation of a particular behaviour [22], ‘knowledge’ about air pollution and health, and ‘behavioural intention,’ which was considered to refer to the degree to which a person is inclined to engage in a particular behaviour [22].

To focus on the scales measuring perception/awareness of air pollution and its associated risks, we selected studies that used ‘validated scales,’ which have been previously tested for reliability and validity, or those that were used more than once in previous studies, so that comparisons between the studies were possible.

We synthesised the data descriptively and presented the results in the form of tables.

## Results

**Figure 1.**
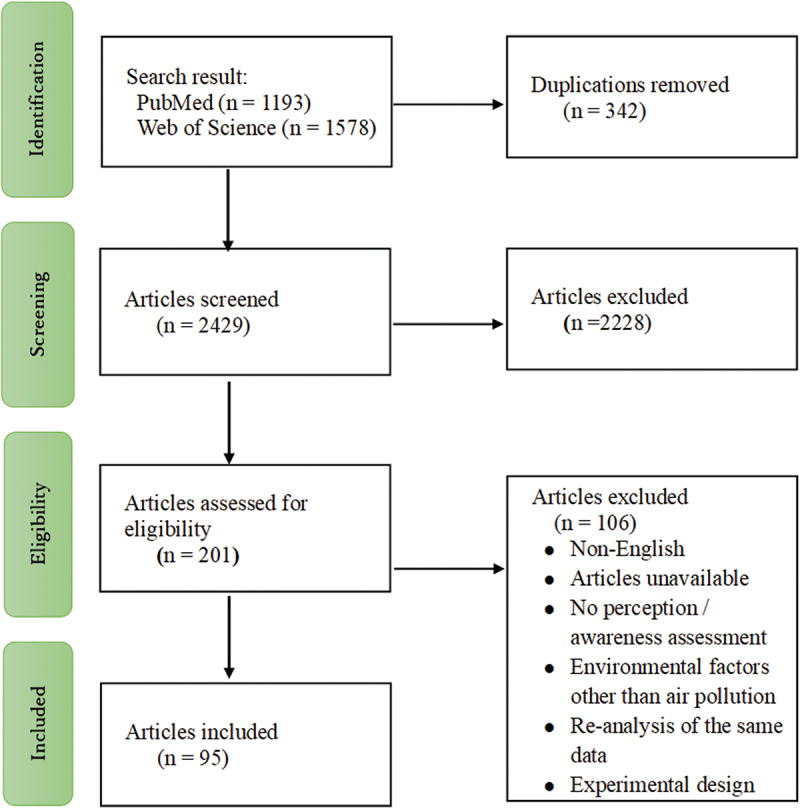
Flow diagram for the process of article selection.

The search identified 1,193 articles in PubMed and 1,578 articles in Web of Science Figure 1 presents a flow diagram of the selection process. After removing the duplication (*n* = 342), initial title and abstract screening identified 201 eligible articles. We obtained and reviewed the full-text, and finally included 95 articles in the analysis (Table S1).

## Overview of the included studies

**Figure 2.**
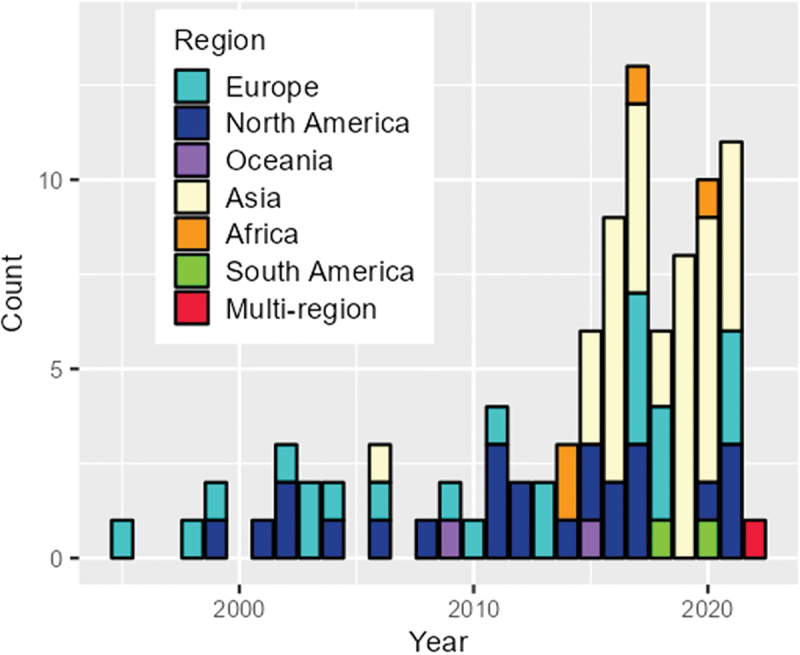
Annual number of articles by region between 1990 and 2022.

Table 1 shows a summary of the included articles. Although the study areas covered six continents, more than half of the studies were conducted in China [23–47] and the United States [21,48–67]. We identified two studies that were conducted in a multi-country setting, namely in seven European countries [68] and in 10 countries across multiple continents during the coronavirus 2019 pandemic; the results revealed that people from different countries were aware of air quality improvement during the implementation of coronavirus disease 2019-related restrictions [69]. The number of articles gradually increased over time, reaching a high of 13 in 2017 (Figure 2). Although most of the earlier studies were conducted in Europe and North America, studies conducted in Asia were most common from the 2010s onwards. We also summarised data for the years in which the surveys were conducted. The time lag from the year of survey until the publication ranged from 0 to 14 years, with a median duration of 3 years (Table 1). However, 10 studies did not clearly report the year of the survey in the manuscript [48,61,70–77].

**Table 1.** Summary of the included studies.

Items		Number of studies (%) (*N* = 95)	
Region of the study^a^			
	Asia	38	(40.0)
	North America	25	(26.3)
	Europe	23	(24.2)
	Africa	4	(4.2)
	South America	2	(2.1)
	Oceania	2	(2.1)
	Across multi-region	1	(1.1)
Publication year			
	− 2000	5	(5.3)
	2001–2005	8	(8.4)
	2006–2010	7	(7.4)
	2011–2015	17	(17.9)
	2016–2020	46	(48.4)
	2021 –	12	(12.6)
Year(s) of survey^b^			
	− 2000	11	(11.6)
	2001–2005	6	(6.3)
	2006–2010	13	(13.7)
	2011–2015	33	(34.7)
	2016–2020	21	(22.1)
	2021 –	1	(1.1)
	Not clearly mentioned	10	(10.5)
Study subjects			
	Residents of the study areas	61	(64.2)
	Nationwide/Online panel	11	(11.6)
	School-related subjects (e.g. students, alumni)	7	(7.4)
	Specific occupational workers	5	(5.3)
	Patients/Physicians	5	(5.3)
	Pedestrians	2	(2.1)
	Others	4	(4.2)
Age			
	Adult	78	(82.1)
	Adult/Adolescent	8	(8.4)
	Adolescent	1	(1.1)
	Adolescent/Children	3	(3.2)
	Children	1	(1.1)
	All age	4	(4.2)
Sex			
	Both sexes	77	(81.1)
	Only male	2	(2.1)
	Only female	1	(1.1)
	Not described	15	(15.8)
Sample size			
	n < 100	9	(9.5)
	100 ≤ *n* < 1000	55	(57.9)
	1000 ≤ *n* < 5000	26	(27.4)
	5000 ≤ n	5	(5.3)
Data collection methods			
	Self-administered questionnaire (In-person, postal, online)	54	(56.8)
	Interview (In-person, online, telephone)	30	(31.6)
	Focus group discussion	2	(2.1)
	Others (‘draw-and write’)	1	(1.1)
	Combined	8	(8.4)
	Interview & Questionnaire	5	(5.3)
	Interview & FDG	2	(2.1)
	Questionnaire & FDG	1	(1.1)

^a^The region of the study was classified according to the definition of ‘Our World in Data’.

^b^If the survey was conducted in the multiple year, the starting year was presented.

^c^‘Others’ include bicycle commuters, sport event participants, runners, both residents and pedestrians.

## Study participants

Table 1 also presents the information on study participants. Sixty-one studies targeted the residents of the study areas [24,27,28,30–34,37,38,41,43–45,48–50,52,53,56–60,62,63,65,69–73,76,78–105] and 11 studies recruited participants using nationwide sampling or online panels [21,35,39,40,42,66–68,106–108]. Several studies targeted school-related subjects [18,26,36,47,61,109,110] and specific occupations workers [29,46,64,74,111].

Although the subjects of most of the studies were adults aged 18 and over, eight studies included both adults and adolescents aged 14–17 years old [30, 36, 39, 41, 82, 84, 89, 102]. Five studies focused on adolescents and children [44,66,67,79,88]. Seventy-seven studies included both men and women, two studies included only men [64,74], one study included only women [71], and 15 studies did not report the proportion of men/women [27,32,45,46,50,51,59,65,70,78,79,83,86,105,110].

## Methods of data collection

The methods for collecting the individual information on the concepts related to health risks from air pollution included self-administered questionnaires either in-person or via online services [18,21,26,28,29,31,36,37, 39–45,47,49,55,60,61,63,65–69,72,74,76,78,82–84,89,94, 98–105,107–116], interviews that were either conducted in-person, via telephone, or via online services [27,30,32–34,46,48,50,52–54,56, 58, 59, 62, 64, 71, 75, 80, 81, 86, 87, 90, 91, 93, 95, 97, 106, 117, 118], focus group discussions [57,92], and a combination of any two of the above [24,38,51,70,73,79,85,96] (Table 1). One study used the ‘draw and write’ technique for children [88], in which respondents drew pictures and wrote responses in relation to specific questions.

## Dimension of health risk perception measured

On the basis of the terms used in the articles, we extracted the key dimensions of perception related to health risks from air pollution (Table 2). Questions about perception/awareness of air quality were observed with the highest frequency, followed by questions about the perception of health risk, knowledge, behavioural intention, and attitude.

**Table 2.** Key dimensions of health risk perception from air pollution.

Dimensions (Multiple choice allowed)	Number of studies
Perception/awareness of air quality	56
Perception of health risk	55
Knowledge	38
Behavioural intention	24
Attitude	20

## Scales of risk perception measuring various dimensions

Among these 95 studies, validated scales of risk perception were used in only nine articles (Table 3), which included studies from China [35,41,44,96], the United States [58,59], Croatia [89], Mexico [97], and France [91]. Most of the scales [39,44,58,97] were specific to each study site.

**Table 3.** Studies that used scaled questionnaires.

First author (year)/country	Study objectives	Name of the questionnaire	Number of items	Concept evaluated	Questions measuring the health risk perception (The items are explained when the specific questions are not available)	scale
Borbet (2018)/Mexico city and state of Mexico, Mexico	To assess knowledge and awareness about daily air quality, and behaviour response.	BRFSS (US 2005 Behaviour Risk Factor Surveillance Systems)	4	Behaviour to reduce exposure, awareness including knowledge of Air Quality Index	1. ‘How many times did you reduce or change your outdoor activity level because you thought the air quality was bad or was affecting how well you felt?’ 2. ‘Have you ever heard or read about the government’s air quality index or air quality alerts where you live?’ 3. ‘How many times did you reduce or change your outdoor activity level based on the air quality index or air quality alerts?’ 4. ‘Has a doctor, nurse, or other health professional ever told you to reduce your outdoor activity level when the air quality is bad?’.	binary
Fleury-Bahi (2013)/Bretagne, Provence-Alpes, Cote d’Azur and Rhone-Alpes, France	To investigate the impact on perceived health and perceived QOL of being exposed to ambient air pollution	WHOQOL-BREF scale	2	Perceived quality of life, perceived health risk due to air pollution	Perceived Health (PH) asking ‘How satisfied are you with your health’ Perceived Quality of Life(PQOL) asking ‘How would you rate your quality of life?’ ‘Have you got, or have you had, health problems due, in your opinion, to ambient air pollution in your neighbourhood?’	5-point Likert into binary for logistic regression
Huang (2018)/China	To examine how issue salience, environmental value, risk perception, and affective response influence information seeking, objective knowledge, and policy support related to this issue.	NA (Adapted from the previous study of risk of climate change)	10	Perceived health risk, attitude (negative affect), behaviour to seek information and to policy support, knowledge	The following questions from the previous studies were adapted: 1. ‘How much do you think climate change will harm (perceived susceptibility) you and your family? Your local community? The United States as a whole? People all over the world? Nature (not including humans)?’ 2. ‘How serious is the threat to you posed by climate change?’, ‘How serious of a threat is climate change to your local community?’, ‘How serious of a threat is climate change to the United States as a whole?’, ‘How serious of a threat is climate change to people all over the world?’, ‘How serious a threat is climate change to nature?’	5–10 point Likert
King (2015)/Chicago, USA	To investigate perceptions of residential outdoor air quality	Community Survey portion of the questionnaire in Chicago Community Adult Health Study (CCAHS)	1	Air pollution perception	‘How would you rate the quality of the air in this neighbourhood?’	4-point Likert
Laws (2015)/Somerville, Dorchester, South Boston & Chinatown, Boston, USA	To assess lay perceptions of risk from air pollution	previously validated questionnaire (Perceived Stress Scale, Multi-Dimensional Health Locus of Control Scale (MHLC)) were used.	1	Perceived Stress Scale	1. ‘How harmful to you believe that air pollution is in this neighbourhood to you or to others who live here?’ 2. ‘In your opinion, should the government do more to protect people in your neighbourhood from air pollution, is the government regulating too much already, or is current policy about right?’ 3. ‘If I become sick, I have the power to make myself well again.’ 4. ‘Often I feel I have no control over whether I will get sick’ 5. ‘It seems my health is greatly influenced by accident’ 6. ‘I am directly responsible for my health’ 7. ‘Whatever goes wrong with my health is my own fault’ 8. ‘When I stay healthy, I am just plain lucky’ 9. ‘When I feel ill, it is because I have not been taking care of myself properly’	4-point Likert 3-point Likert
Pu (2019)/Nationwide, China	To provide a nationwide view on the public’s air pollution risk perception and attitude.	(questionnaire based on psychometric paradigm method)	20	Risk perception, knowledge, Attitude	1. Risk perception, consisting of 6 factors (risk benefit, environmental awareness, knowledge about air pollution, perceived risk of air pollution, avoidance of air pollution, trust of individual ability and government air pollution policies),	5-point Likert
Valentic (2010)/Kostrena and Crikvenica, Croatia	To compare the perception of health of the population between the areas with difference air pollution levels	36-Item Short-Form Health Survey (SF-36)	35	Perceived health	1. Physical functioning, 2. Role limitation due to physical problems 3. Role limitation due to emotional problems, 4. Social functioning, 5. Mental health, 6. Vitality and energy, 7. Bodily pain, 7General health perception	0–100 score
Zhao (2020)/Baoding, China	To investigate the level of haze-related knowledge	AHRKAAS (Adolescent Haze-Related Knowledge Awareness Assessment Scale)	25	Knowledge and awareness about haze, its health risks, and protection measures	4 Dimensions with 25 items (‘the cognition f human factors of haze formation’, ‘The cognition of natural factors of haze formation’, ‘The cognition of haze harmful effects on the human body’, and ‘The cognition of haze health protection measures’	5-point Likert scale with total score ranging 25–125
Zhou (2019)/Beijing, Shanghai, and Guanzhou, China	To explore the influencing factors of haze tolerance	The questionnaire adapted from the studies of other environmental hazard, such as nuclear accident and wind power	23	Tolerance (4 items), political trust (4 items), risk perception (7 items), cost perception (3 items), knowledge (5 items)	1. ‘I’m worried about the health hazards caused by haze’ 2. “I think haze will frequently appear in Beijing/Shanghai/Guangzhou and endanger people’s health, 3. I will have a shorter life due to haze during my stay in Beijing/Shanghai/Guangzhou, 4. I’m very scared of the haze in Beijing/Shanghai/Guangzhou, 5 My families and friends will suffer from respiratory diseases, asthma, cardiovascular and cerebrovascular diseases, and cancer due to haze, 6. I think haze is more harmful to human health than smoking	5-point Likert scale

Abbreviation: AHRKAAS: Adolescent Haze-Related Knowledge Awareness Assessment Scale; BRFSS: Behavior Risk Factor Surveillance Systems; CCAHS: Chicago Community Adult Health Study; IMECA: ÍndiceMetropolitano de la Calidad del Aire [Metropolitan Index of Air Quality]; WHOQOL-BREF: World Health Organisation Quality of Life-Brief.

We summarised individual scales (Table 3, Table S2) and focused on the dimensions evaluated in each scale in the process from perception of air pollution to perception of health risks and subsequent behaviour to avoid those risks (Table 4). Among the nine studies mentioned above, perception of health risk was the most frequently evaluated dimension [35,39,41,59,89,91]. A few studies [35,41] asked the health risk posed by air pollution using the validated questionnaires adapted from the previous studies to assess risk perception of other environmental hazards, such as climate change [119] and nuclear power [120,121]. WHOQOL-BREF and SF-36, measuring perceived health and QOL, do not necessarily inquire about health risk perception related to air pollution. Thus, additional questions regarding air pollution were included in the studies [89,91]. Three studies using scales to measure the dimensions of attitude [35,39,41], the dimension of knowledge was also measured. The content and quantity of questions measuring the dimension of knowledge vary across studies. While AHRKAAS has 25 detailed items that ask the knowledge about various aspects of air pollution (haze), from its sources to health impacts [44], BRFSS focused on the knowledge of the air quality index/alert, which could lead to behavioural response [97]. There are two scales measuring behaviour dimensions [35,97], and the questions concerning specific behaviours differed between them. One scale asked the behaviours to reduce outdoor activities to avoid air pollution [97] while other measured information seeking behaviours [35].

**Table 4.** The dimensions measured by scaled studies.

Questionnaire	Perception of health risk	Perception/ awareness of air quality	Attitude	Knowledge	Behavioural intention	Author (publication year)
BRFSS (US 2005 Behavior Risk Factor Surveillance Systems)				✓	✓	Borbet et al. (2018)
WHOQOL-BREF scale	✓					Fleury-Bahi et al. (2013)
Adapted from the previous study of risk of climate change	✓		✓	✓	✓	Huang et al. (2018)
Community Survey portion of the questionnaire in Chicago Community Adult Health Study (CCAHS)		✓				King (2015)
Previously validated questionnaire (Perceived Stress Scale, Multi-Dimensional Health Locus of Control Scale (MHLC))	✓					Laws et al. (2015)
(questionnaire based on psychometric paradigm method)	✓		✓	✓		Pu et al. (2019)
SF-36	✓					Valentic et al. (2010)
AHRKAAS (Adolescent Haze-Related Knowledge Awareness Assessment Scale)				✓		Zhao et al. (2020)
Adapted from the studies of other environmental hazard (nuclear accident and wind power)	✓		✓	✓		Zhou et al. (2019)

## Discussion

In the current scoping review, we identified 95 studies evaluating perceptions of the risk related to air pollution. The number of studies focusing on the perception of air pollution and its health risks has been increasing over time. Since the mid-2010s, the majority of studies investigating this topic have been conducted in Asia, led by China. This may be at least in part because air pollution emissions dramatically increased in these regions with rapid industrialisation and urbanisation. Additionally, media reporting on the extremely high levels of air pollution levels in some parts of Asia increased public concern about health risk from air pollutants. Air pollution is not only a local issue, but also a global issue. Recently, several studies have been conducted in Africa [73,92,96,103] and South America [97,104] where rapid economic development is advancing (Table 1, Figure 2). The study areas are generally affected by air pollutants from heavy traffic and odorous industrial emissions, and most of the studies have emphasised the importance of knowledge about air quality [73,92,97,103]. Global-scale studies using standardised scales to measure the perception of air pollution and its health risks are essential for elucidating the common factors affecting individuals’ perceptions regarding air quality and its health risks. However, few studies have been conducted across multiple countries.

## Standardised scales

Only nine studies used standardised scales for which validity and reliability has been evaluated and tested [35,41,44,58,59,89,91,96,97]. There is considerable variation among standardised scales for measuring the multiple dimensions of risk perception for air pollution, and none of the studies in the present review used the same scales. Table 4 illustrated the dimensions measured within each study’s scale, including the perception of health risk, perception/awareness of air quality, attitudes, knowledge, and behavioural intention. However, the response indicators used different scales, including binary variables and 3–5-point Likert scales. Additionally, the terms used in the scale vary among the studies, which makes comparisons among studies difficult. Although the objectives vary between studies, using a standardised scale may facilitate understanding of the process of health behavioural change. This may also elucidate the local attributes that influence risk perception when there are regional differences in the risk perception of air pollution. Standardised scales are even more important in studies conducted to evaluate the effectiveness of interventions. Although WHOQOL-BREF and SF-36 have been used in the field of public health, they are not necessarily used to examine perceived health related to air pollution. Most of the available scales, except for WHOQOL-BREF and SF-36, were developed in a local context, assuming a specific source of pollution in the area. For example, the BRFSS and CCAHS focused on local air pollution. In a study conducted in China [35], participants were asked how they perceived the seriousness of air pollution not only for the local community but also for the whole country, and for the world. Regarding these questions, it can be assumed that the researchers themselves see air pollution as a wider regional or global problem rather than a local issue. Creating a scale that can be used at the global scale to measure perceptions of air pollution worldwide may facilitate the comparison of the results among studies.

## Scale for children

Fewer than 15% of the studies included in the current review involved adolescents and children (Table 1). Children are more susceptible to air pollution than adults. Improving children’s health literacy is considered to be increasingly important. WHOQOL-BREF and SF-36 have been used in studies of perceived QOL for adults. A standardised scale has also been developed for measuring perceived QOL among children. In this review, we did not identify any studies that measured the perceived QOL for children using a standardised scale, such as the Paediatric Quality of Life Inventory [122]. The AHRKAAS has been used in China [44] with a focus on adolescents, because adolescents and children are considered to be more susceptible to air pollution than adults. This scale was initially developed in China, and evaluates knowledge about emission sources, health effects, and health protection measures [123]. The authors discussed the limitations of the generalisability of this scale because the validation study was conducted in a single city. Further validation studies will be necessary in international settings.

## Dimensions of perception

We summarised the key dimensions of perceptions related to health risks from air pollution. Both ‘Perception/awareness of air quality’ and ‘Perception of health risk’ were the most frequently evaluated dimensions of perception. Specifically, we found that the largest number of studies focusing on ‘Perception/awareness of air quality’ during the mid to late 2010s were conducted in Asia. This result may reflect that poor air quality was often visually perceived during this period in the study areas, and that the media provided more coverage about air pollution. Surprisingly, several studies did not report when the survey was conducted. The study period is one of the most important factors affecting perception, because perception of air quality and its health risks are affected by the air quality levels at the time the survey is conducted. Additionally, environmental regulations also affect people’s perceptions.

## Limitations

Several limitations of this scoping review should be considered. This review did not include non-English articles, unpublished articles, or grey literature. Regarding studies published before the 2010s, it is likely that some studies evaluating perceived health risk from air pollution were published in non-English languages, because air pollution was considered as a local issue. This may have influenced the results of the trends we observed in the region-specific categorisation. Additionally, there was considerable variation in the terms used to describe the perceived health risks related to air pollution. This makes the interpretation of the results difficult. To overcome this limitation, we extracted studies using validated scales.

## Conclusion

In this scoping review, we explored published articles investigating the perception of health risks posed by air pollution, focusing on the measurement scales used. Only nine studies used standardised scales to quantify individuals’ risk perception. Spatial and temporal comparisons of the health risk perception of air pollution may facilitate the understanding of individuals’ behaviours to prevent air pollution exposure and support air pollution policies. Thus, further development of standardised methods is needed.

## Supplementary Material

SupplementalTable1_2.xlsx

Figure2.tif

## References

[1] Slovic P. Perception of risk. Science. 1987;236:280–13. doi: 10.1126/science.3563507 3563507

[2] Ferrer RA, Klein WMP. Risk perceptions and health behavior. Curr Opin Psychol. 2015;5:85–89. doi: 10.1016/j.copsyc.2015.03.012 26258160 PMC4525709

[3] Brewer NT, Chapman GB, Gibbons FX, et al. Meta-analysis of the relationship between risk perception and health behavior: the example of vaccination. Health Psychol. 2007;26:136–145. doi: 10.1037/0278-6133.26.2.136 17385964

[4] Cahyanto I, Wiblishauser M, Pennington-Gray L, Schroeder A, et al. The dynamics of travel avoidance: the case of Ebola in the U.S. Tour Manag Perspect. 2016;20:195–203. doi: 10.1016/j.tmp.2016.09.004 32289007 PMC7147605

[5] Bruine de Bruin, de Bruin Wb D, Bruine de Bruin W, et al. Relationships between initial COVID-19 risk perceptions and protective health behaviors: A national survey. Am J Prev Med. 2020;59:157–167. doi: 10.1016/j.amepre.2020.05.001 32576418 PMC7242956

[6] Bubeck P, Botzen WJW, Jcjh A, et al. A review of risk perceptions and other factors that influence flood mitigation behavior. Risk Anal. 2012;32:1481–1495. doi: 10.1111/j.1539-6924.2011.01783.x 22394258

[7] Schneiderbauer S, Pisa PF, Delves JL, et al. Risk perception of climate change and natural hazards in global mountain regions: a critical review. Sci Total Environ. 2021;784:146957. doi: 10.1016/j.scitotenv.2021.146957 33895507

[8] Takebayashi Y, Lyamzina Y, Suzuki Y, et al. Risk perception and anxiety regarding radiation after the 2011 Fukushima nuclear power plant accident: a systematic qualitative review. Int J Environ Res Public Health. 2017;14:1306. doi: 10.3390/ijerph14111306 29077045 PMC5707945

[9] Ban J, Shi WY, Cui LL, et al. Health-risk perception and its mediating effect on protective behavioral adaptation to heat waves. Environ Res. 2019;172:27–33. doi: 10.1016/j.envres.2019.01.006 30769186

[10] Xu ZH, Li JM, Shan JZ, et al. Extending the theory of planned behavior to understand residents’ coping behaviors for reducing the health risks posed by haze pollution. Environ Dev Sus. 2021;23:2122–2142. doi: 10.1007/s10668-020-00666-5

[11] Dietz T, Dan A, Shwom R, et al. Support for climate change policy: social psychological and social structural influences. Rural Sociol. 2007;72:185–214. doi: 10.1526/003601107781170026

[12] Boso A, Hofflinger AQ, Oltra C, et al. Public support for wood smoke mitigation policies in south-central Chile. Air Qual Atmos Health. 2018;11:1109–1119. doi: 10.1007/s11869-018-0612-2

[13] Pidgeon N. Risk assessment, risk values and the social science programme: why we do need risk perception research. Reliab Eng Syst Saf. 1998;59:5–15. doi: 10.1016/S0951-8320(97)00114-2

[14] Bickerstaff K. Risk perception research: socio-cultural perspectives on the public experience of air pollution. Environ Int. 2004;30:827–840. doi: 10.1016/j.envint.2003.12.001 15120202

[15] Renn O. The role of risk perception for risk management. Reliab Eng Syst Saf. 1998;59:49–62. doi: 10.1016/S0951-8320(97)00119-1

[16] Cori L, Donzelli G, Gorini F, et al. Risk perception of air pollution: a systematic review focused on particulate matter exposure. Int J Environ Res Public Health. 2020;17:6424. doi: 10.3390/ijerph17176424 32899325 PMC7504632

[17] Ban J, Zhou L, Zhang Y, et al. The health policy implications of individual adaptive behavior responses to smog pollution in urban China. Environ Int. 2017;106:144–152. doi: 10.1016/j.envint.2017.06.010 28651244

[18] Lin TT, Li L, Bautista JR, et al. Examining how communication and knowledge relate to Singaporean youths’ perceived risk of haze and intentions to take preventive behaviors. Health Commun. 2017;32:749–758. doi: 10.1080/10410236.2016.1172288 27392280

[19] Arksey H, O’Malley L. Scoping studies: towards a methodological framework. Int J Soc Res Method. 2005;8:19–32. doi: 10.1080/1364557032000119616

[20] Levac D, Colquhoun H, O’Brien KK, et al. Scoping studies: advancing the methodology. Implement Sci. 2010;5:69. doi: 10.1186/1748-5908-5-69 20854677 PMC2954944

[21] Mirabelli MC, Ebelt S, Damon SA, et al. Air quality index and air quality awareness among adults in the United states. Environ Res. 2020;183:109185. doi: 10.1016/j.envres.2020.109185 32007750 PMC7182097

[22] Ajzen I. The theory of planned behavior. Organ Behav Hum Dec. 1991;50:179–211. doi: 10.1016/0749-5978(91)90020-T

[23] Liao X, Tu H, Maddock JE, et al. Residents’ perception of air quality, pollution sources, and air pollution control in Nanchang, China. Atmos Pollut Res. 2015;6:835–841. doi: 10.5094/apr.2015.092

[24] Zhang AP, Zhong LS, Xu Y, et al. Tourists’ perception of haze pollution and the potential impacts on travel: reshaping the features of tourism seasonality in Beijing, China. Sustainability-Basel. 2015;7:2397–2414. doi: 10.3390/su7032397

[25] Guo YL, Liu FF, Lu YA, et al. Factors affecting parent’s perception on air quality from the individual to the community level. Int J Environ Res Public Health. 2016;13:493. doi: 10.3390/ijerph13050493 27187432 PMC4881118

[26] Lan GL, Yuan ZK, Maddock JE, et al. Public perception of air pollution and health effects in Nanchang, China. Air Qual Atmos Health. 2016;9:951–959. doi: 10.1007/s11869-016-0397-0

[27] Li Z, Folmer H, Xue J, et al. Perception of air pollution in the Jinchuan mining Area, China: a structural equation modeling approach. Int J Environ Res Public Health. 2016;13:735. doi: 10.3390/ijerph13070735 27455291 PMC4962276

[28] Liu X, Zhu H, Hu Y, et al. Public’s health risk awareness on urban air pollution in Chinese megacities: the cases of Shanghai, Wuhan and Nanchang. Int J Environ Res Public Health. 2016b;13:845. doi: 10.3390/ijerph13090845 27571088 PMC5036678

[29] Liu X, Wu Y, Hu Y, et al. Government employees’ perception of urban air pollution and willingness to pay for improved quality: a cross-sectional survey study in Nanchang, China. Environ Sci Pollut Res. 2016a;23:22183–22189. doi: 10.1007/s11356-016-7204-1 27562814

[30] Qian X, Xu G, Li L, et al. Knowledge and perceptions of air pollution in Ningbo, China. BMC Public Health. 2016;16:1138. doi: 10.1186/s12889-016-3788-0 27816059 PMC5097845

[31] Huang L, Rao C, van der Kuijp Tj, et al. A comparison of individual exposure, perception, and acceptable levels of PM(2.5) with air pollution policy objectives in China. Environ Res. 2017;157:78–86. doi: 10.1016/j.envres.2017.05.012 28525860

[32] Tvinnereim E, Liu XZ, Jamelske EM, et al. Public perceptions of air pollution and climate change: different manifestations, similar causes, and concerns. Clim Change. 2017;140:399–412. doi: 10.1007/s10584-016-1871-2

[33] Xu JH, Chi CSF, Zhu K, et al. Concern or apathy: the attitude of the public toward urban air pollution. J. Risk Res. 2017;20:482–498. doi: 10.1080/13669877.2015.1071869

[34] Yang S, Shi L. Public perception of smog: a case study in Ningbo City, China. J Air Waste Manag Assoc. 2017;67:219–230. doi: 10.1080/10962247.2016.1229235 27629231

[35] Huang JL, Yang ZJ. Risk, affect, and policy support: public perception of air pollution in China. Asian J Commun. 2018a;28:281–297. doi: 10.1080/01292986.2017.1386220

[36] Rajper SA, Ullah S, Li Z, et al. Exposure to air pollution and self-reported effects on Chinese students: a case study of 13 megacities. PLOS One. 2018;13:e0194364. doi: 10.1371/journal.pone.0194364 29547657 PMC5856349

[37] Chen S, Qin P, Tan-Soo JS, et al. Recency and projection biases in air quality valuation by Chinese residents. Sci Total Environ. 2019;648:618–630. doi: 10.1016/j.scitotenv.2018.08.153 30121539

[38] Li XY, Tilt B. Public engagements with smog in urban China: Knowledge, trust, and action. Environ Sci Policy. 2019;92:220–227. doi: 10.1016/j.envsci.2018.12.008

[39] Pu S, Shao Z, Fang M, et al. Spatial distribution of the public’s risk perception for air pollution: A nationwide study in China. Sci Total Environ. 2019;655:454–462. doi: 10.1016/j.scitotenv.2018.11.232 30472647

[40] Wang SY, Wang J, Ru XJ, et al. Public smog knowledge, risk perception, and intention to reduce car use: evidence from China. Hum Ecol Risk Assess. 2019;25:1745–1759. doi: 10.1080/10807039.2018.1471580

[41] Zhou L, Dai Y. The influencing factors of haze tolerance in China. Int J Environ Res Public Health. 2019;16:287. doi: 10.3390/ijerph16020287 30669599 PMC6352209

[42] Huang Q. How does news media exposure amplify publics’ perceived health risks about air pollution in China? A conditional media effect approach. Int J Commun-US. 2020;14:1705–1724.

[43] Yang QH, Wu SW. Air pollution in China: health information seeking and protective behaviors. Health Promt Int. 2020;35:1495–1506. doi: 10.1093/heapro/daaa017 32211759

[44] Zhao Q, Zhao Y, Dou H, et al. Adolescent haze-related knowledge level study: a cross-sectional survey with sensitivity analysis. Front Public Health. 2020;8:229. doi: 10.3389/fpubh.2020.00229 32733831 PMC7363765

[45] Zhu WW, Yao NZ, Guo QZ, et al. Public risk perception and willingness to mitigate climate change: city smog as an example. Environ Geochem Health. 2020;42:881–893. doi: 10.1007/s10653-019-00355-x 31227948

[46] Winter AK, Le H, Roberts S, et al. From black to blue skies: civil society perceptions of air pollution in Shanghai. China Quart. 2021;248:1059–1080. doi: 10.1017/S0305741021000588

[47] Zhou Q, Chen N, Pan X, et al. Characterizing air pollution risk perceptions among high-educated young generation in China: How does risk experience influence risk perception. Environ Sci Policy. 2021;123:99–105. doi: 10.1016/j.envsci.2021.05.006

[48] Creer RN, Gray RM, Treshow M, et al. Differential responses to air pollution as an environmental health problem. J Air Pollut Control Assoc. 1970;20:814–818. doi: 10.1080/00022470.1970.10469478 5515469

[49] Johnson BB. Gender and race in beliefs about outdoor air pollution. Risk Anal. 2002;22:725–738. doi: 10.1111/0272-4332.00064 12224746

[50] Brody SD, Peck BM, Highfield WE, et al. Examining localized patterns of air quality perception in Texas: a spatial and statistical analysis. Risk Anal. 2004;24:1561–1574. doi: 10.1111/j.0272-4332.2004.00550.x 15660612

[51] McDermott M, Srivastava R, Croskell S, et al. Awareness of and compliance with air pollution advisories: a comparison of parents of asthmatics with other parents. J Asthma. 2006;43:235–239. doi: 10.1080/02770900600567114 16754528

[52] Semenza JC, Wilson DJ, Parra J, et al. Public perception and behavior change in relationship to hot weather and air pollution. Environ Res. 2008;107:401–411. doi: 10.1016/j.envres.2008.03.005 18466894

[53] Johnson BB. Acculturation, ethnicity, and air pollution perceptions. Risk Anal. 2011;31:984–999. doi: 10.1111/j.1539-6924.2010.01557.x 21231941

[54] Nikolopoulou M, Kleissl J, Linden PF, et al. Pedestrians’ perception of environmental stimuli through field surveys: focus on particulate pollution. Sci Total Environ. 2011;409:2493–2502. doi: 10.1016/j.scitotenv.2011.02.002 21492905

[55] Nowka MR, Bard RL, Rubenfire M, et al. Patient awareness of the risks for heart disease posed by air pollution. Prog Cardiovasc Dis. 2011;53:379–384. doi: 10.1016/j.pcad.2010.12.003 21414473

[56] Johnson BB. Experience with urban air pollution in Paterson, new jersey and implications for air pollution communication. Risk Anal. 2012;32:39–53. doi: 10.1111/j.1539-6924.2011.01669.x 21883333

[57] Kondo MC, Gross-Davis CA, May K, et al. Place-based stressors associated with industry and air pollution. Health Place. 2014;28:31–37. doi: 10.1016/j.healthplace.2014.03.004 24721738 PMC4065639

[58] King KE. Chicago residents’ perceptions of air quality: objective pollution, the built environment, and neighborhood stigma theory. Popul Environ. 2015;37:1–21. doi: 10.1007/s11111-014-0228-x 26527847 PMC4627697

[59] Laws MB, Yeh Y, Reisner E, et al. Gender, ethnicity and environmental risk perception revisited: the importance of residential location. J Community Health. 2015;40:948–955. doi: 10.1007/s10900-015-0017-1 25822317 PMC4558345

[60] Brown P, Cameron L, Cisneros R, et al. Latino and non-latino perceptions of the air quality in california’s san joaquin valley. Int J Environ Res Public Health. 2016;13:1242. doi: 10.3390/ijerph13121242 27983706 PMC5201383

[61] Zhou Y, Song Y, Tian J, et al. Risk perception of air pollution: an exploration of self-relevancy. Hum Ecol Risk Assess. 2016;22:1506–1518. doi: 10.1080/10807039.2016.1190635

[62] Chakraborty J, Collins TW, Grineski SE, et al. Racial differences in perceptions of air pollution health risk: does environmental exposure matter? Int J Environ Res Public Health. 2017;14:116. doi: 10.3390/ijerph14020116 28125059 PMC5334670

[63] Cisneros R, Brown P, Cameron L, et al. Understanding public views about air quality and air pollution sources in the san joaquin valley, California. J Environ Public Health. 2017;2017:4535142. doi: 10.1155/2017/4535142 28469673 PMC5392406

[64] Gany F, Bari S, Prasad L, et al. Perception and reality of particulate matter exposure in New York City taxi drivers. J Expo Sci Env Epid. 2017;27:221–226. doi: 10.1038/jes.2016.23 PMC5547750 27168392

[65] Benney TM, Cantwell D, Singer P, et al. Understanding perceptions of health risk and behavioral responses to air pollution in the state of Utah (USA). Atmosphere. 2021;12:1373. doi: 10.3390/atmos12111373

[66] Lynch KM, Mirabelli MC. Air quality awareness and behaviors of US adolescents with and without asthma. Am J Prev Med. 2021a;61:724–728. doi: 10.1016/j.amepre.2021.04.030 34229930 PMC8541932

[67] Lynch KM, Mirabelli MC. Outdoor air quality awareness, perceptions, and behaviors among U.S. children aged 12-17 years, 2015-2018. J Adolesc Health. 2021b;68:882–887. doi: 10.1016/j.jadohealth.2020.07.040 32919887 PMC7940452

[68] Maione M, Mocca E, Eisfeld K, et al. Public perception of air pollution sources across Europe. Ambio. 2021;50:1150–1158. doi: 10.1007/s13280-020-01450-5 33382442 PMC8068740

[69] Lou BW, Barbieri DM, Passavanti M, et al. Air pollution perception in ten countries during the COVID-19 pandemic. Ambio. 2022;51:531–545. doi: 10.1007/s13280-021-01574-2 34155609 PMC8216327

[70] Stevens E, Cullinan P, Colvile R, et al. Urban air pollution and children’s asthma: what do parents and health professionals think? Pediatr Pulmonol. 2004;37:530–536. doi: 10.1002/ppul.20008 15114554

[71] Edgley A, Pilnick A, Clarke M, et al. ‘The air still wasn’t good … everywhere I went I was surrounded’: lay perceptions of air quality and health. Health Sociol Rev. 2011;20:97–108. doi: 10.5172/hesr.2011.20.1.97

[72] Claeson AS, Lidén E, Nordin M, et al. The role of perceived pollution and health risk perception in annoyance and health symptoms: a population-based study of odorous air pollution. Int Arch Occup Environ Health. 2013;86:367–374. doi: 10.1007/s00420-012-0770-8 22526088

[73] Omanga E, Ulmer L, Berhane Z, et al. Industrial air pollution in rural Kenya: community awareness, risk perception and associations between risk variables. BMC Public Health. 2014;14:377. doi: 10.1186/1471-2458-14-377 24742166 PMC4012528

[74] Wannalai S, Nokaew S, Siriwong W, et al. Assessment of knowledge and perception of adverse health effects associated with self-prevention from air pollution in traffic policemen in Bangkok, Thailand. J Health Res. 2016;30:S147–S52. doi: 10.14456/jhr.2016.78

[75] Hodgson A, Hitchings R. Urban air pollution perception through the experience of social practices: Talking about breathing with recreational runners in London. Health Place. 2018;53:26–33. doi: 10.1016/j.healthplace.2018.07.009 30048828

[76] Yazdanibakhsh F, Salehi E, Faham E, et al. Influential factors of air pollution awareness in Isfahan, Iran. Pollution. 2019;5:247–256. doi: 10.22059/poll.2018.256967.435

[77] Zielonka TM. Awareness of Polish physicians regarding the impact of air pollution on health. Arch Environ Occup Health. 2022;77:478–485. doi: 10.1080/19338244.2021.1935677 34096478

[78] Moffatt S, Phillimore P, Bhopal R, et al. ‘If this is what it’s doing to our washing, what is it doing to our lungs?’ Industrial pollution and public understanding in north-east England. Soc Sci Med. 1995;41:883–891. doi: 10.1016/0277-9536(94)00380-c 8571160

[79] Slachtova H, Tomasek I, Jones K, et al. Risk perception study in the framework of PHARE/CESAR study - central European study on air pollution and respiratory health risk perception, the environment, and communication strategies in the CESAR project: results from the Czech Republic. J Hazard Mater. 1998;61:313–317. doi: 10.1016/s0304-3894(98)00138-1

[80] Elliott SJ, Cole DC, Krueger P, et al. The power of perception: health risk attributed to air pollution in an urban industrial neighbourhood. Risk Anal. 1999;19:621–634. doi: 10.1111/j.1539-6924.1999.tb00433.x 10765426

[81] Wakefield SEL, Elliott SJ, Cole DC, et al. Environmental risk and (re)action: air quality, health, and civic involvement in an urban industrial neighbourhood. Health Place. 2001;7:163–177. doi: 10.1016/s1353-8292(01)00006-5 11439253

[82] Howel D, Moffatt S, Prince H, et al. Urban air quality in north-East England: exploring the influences on local views and perceptions. Risk Anal. 2002;22:121–130. doi: 10.1111/0272-4332.t01-1-00010 12017354

[83] Luginaah IN, Taylor SM, Elliott SJ, et al. Community reappraisal of the perceived health effects of a petroleum refinery. Soc Sci Med. 2002;55:47–61. doi: 10.1016/S0277-9536(01)00206-4 12137188

[84] Howel D, Moffatt S, Bush J, et al. Public views on the links between air pollution and health in Northeast England. Environ Res. 2003;91:163–171. doi: 10.1016/S0013-9351(02)00037-3 12648479

[85] Day RJ. Traffic-related air pollution and perceived health risk: lay assessment of an everyday hazard. Health Risk Soc. 2006;8:305–322. doi: 10.1080/13698570600871869

[86] Walker TR, Habeck JO, Karjalainen TP, et al. Perceived and measured levels of environmental pollution: interdisciplinary research in the subarctic lowlands of northeast European Russia. AMBIO: A J Hum Environ. 2006;35:220–228. doi: 10.1579/06-a-127r.1 16989506

[87] Badland HM, Duncan MJ. Perceptions of air pollution during the work-related commute by adults in Queensland, Australia. Atmos Environ. 2009;43:5791–5795. doi: 10.1016/j.atmosenv.2009.07.050

[88] Pluhar ZF, Piko BF, Kovacs S, et al. “Air pollution is bad for my health”: Hungarian children’s knowledge of the role of environment in health and disease. Health Place. 2009;15:239–246. doi: 10.1016/j.healthplace.2008.05.005 18620887

[89] Valentić D, Micović V, Kolarić B, et al. The role of air quality in perception of health of the local population. Coll Antropol. 2010;34:113–117. 21305731

[90] Simone D, Eyles J, Newbold KB, et al. Air quality in Hamilton: who is concerned? Perceptions from three neighbourhoods. Soc Indic Res. 2012;108:239–255. doi: 10.1007/s11205-012-0064-2

[91] Fleury-Bahi G, Preau M, Annabi-Attia T, et al. Perceived health and quality of life: the effect of exposure to atmospheric pollution. J. Risk Res. 2013;18:127–138. doi: 10.1080/13669877.2013.841728

[92] Muindi K, Egondi T, Kimani-Murage E, et al. “We are used to this”: a qualitative assessment of the perceptions of and attitudes towards air pollution amongst slum residents in Nairobi. BMC Public Health. 2014;14:226. doi: 10.1186/1471-2458-14-226 24597487 PMC3973865

[93] Liao PS, Shaw D, Lin YM, et al. Environmental quality and life satisfaction: subjective versus objective measures of air quality. Soc Indic Res. 2015;124:599–616. doi: 10.1007/s11205-014-0799-z

[94] Carducci A, Donzelli G, Cioni L, et al. Air pollution: a study of citizen’s attitudes and behaviors using different information sources. Ebph. 2017;14:e12389. doi: 10.2427/12389

[95] Deguen S, Padilla M, Padilla C, et al. Do individual and neighborhood characteristics influence perceived air quality? Int J Environ Res Public Health. 2017;14:1559. doi: 10.3390/ijerph14121559 29231899 PMC5750977

[96] Ngo NS, Kokoyo S, Klopp J, et al. Why participation matters for air quality studies: risk perceptions, understandings of air pollution and mobilization in a poor neighborhood in Nairobi, Kenya. Public Health. 2017;142:177–185. doi: 10.1016/j.puhe.2015.07.014 26298585

[97] Borbet TC, Gladson LA, Cromar KR, et al. Assessing air quality index awareness and use in Mexico City. BMC Public Health. 2018;18:538. doi: 10.1186/s12889-018-5418-5 29688852 PMC5913808

[98] Oltra C, Sala R. Perception of risk from air pollution and reported behaviors: a cross-sectional survey study in four cities. J. Risk Res. 2018;21:869–884. doi: 10.1080/13669877.2016.1264446

[99] Chin YSJ, De Pretto L, Thuppil V, et al. Public awareness and support for environmental protection-A focus on air pollution in Peninsular Malaysia. PLOS One. 2019;14:e0212206. doi: 10.1371/journal.pone.0212206 30870439 PMC6417846

[100] Saleem Z, Saeed H, Yousaf M, et al. Evaluating smog awareness and preventive practices among Pakistani general population: a cross-sectional survey. Int J Health Promot Educ. 2019;57:161–173. doi: 10.1080/14635240.2019.1576535

[101] Chang FJ, Ashfold MJ. Public perceptions of air pollution and its health impacts in greater kuala Lumpur. In: IOP Conference Series: Earth and Environmental Science; Kuala Lumpur, Malaysia; 2020;489. p. 012027.

[102] Jokar M, Razavi Z, Moradi H, et al. From environmental knowledge to encouraging pro-environmental behavior for air pollution control in Isfahan: a highly air-polluted city in central Iran. SN Appl Sci. 2020;2:1986. doi: 10.1007/s42452-020-03777-w

[103] Odonkor ST, Mahami T. Knowledge, attitudes, and perceptions of air pollution in Accra, Ghana: A critical survey. J Environ Public Health. 2020;2020:1–10. doi: 10.1155/2020/3657161 PMC7040415 32104187

[104] Valencia ARZ, Umana MR, Wences HJA, et al. The air quality perception of residents in the metropolitan zone of acapulco who live around intersections with intense traffic. Environments. 2020;7:21. doi: 10.3390/environments7030021

[105] Krishna BA, Devi RG, Priya AJ, et al. Knowledge, attitude and awareness on effect of air pollution on children among parents. J Pharm Res Int. 2021;33:347–354. doi: 10.9734/JPRI/2021/v33i47B33134

[106] Riedel N, Loerbroks A, Bolte G, et al. Do perceived job insecurity and annoyance due to air and noise pollution predict incident self-rated poor health? A prospective analysis of independent and joint associations using a German national representative cohort study. BMJ Open. 2017;7:e012815. doi: 10.1136/bmjopen-2016-012815 PMC5278244 28115332

[107] Orru K, Nordin S, Harzia H, et al. The role of perceived air pollution and health risk perception in health symptoms and disease: a population-based study combined with modelled levels of PM(10). Int Arch Occup Environ Health. 2018;91:581–589. doi: 10.1007/s00420-018-1303-x 29602966 PMC6002462

[108] Kim B, Yoon EJ, Kim S, et al. The effects of risk perceptions related to particulate matter on outdoor activity satisfaction in South Korea. Int J Environ Res Public Health. 2020;17:1613. doi: 10.3390/ijerph17051613 32131520 PMC7084646

[109] Ullah S, Ullah N, Rajper SA, et al. Air pollution and associated self-reported effects on the exposed students at Malakand division, Pakistan. Environ Monit Assess. 2021;193:708. doi: 10.1007/s10661-021-09484-2 34623541 PMC8498981

[110] Beaumont R, Hamilton RS, Machin N, et al. Social awareness of air quality information. Sci Total Environ. 1999;235:319–329. doi: 10.1016/s0048-9697(99)00215-6 10535126

[111] Al-Shidi HK, Ambusaidi AK, Sulaiman H, et al. Public awareness, perceptions and attitudes on air pollution and its health effects in Muscat, Oman. J Air Waste Manag Assoc. 2021;71:1159–1174. doi: 10.1080/10962247.2021.1930287 33989134

[112] Cole-Hunter T, Morawska L, Solomon C, et al. Bicycle commuting and exposure to air pollution: a questionnaire-based investigation of perceptions, symptoms, and risk management strategies. J Phys Act Health. 2015;12:490–499. doi: 10.1123/jpah.2013-0122 24904984

[113] De Pretto L, Acreman S, Ashfold MJ, et al. The link between knowledge, attitudes and practices in relation to atmospheric haze pollution in Peninsular Malaysia. PLOS One. 2015;10:e0143655. doi: 10.1371/journal.pone.0143655 26646896 PMC4672926

[114] Guo Y, Liu F, Lu Y, et al. Factors affecting parent’s perception on air quality—from the individual to the community level. Int J Environ Res Public Health. 2016;13:493. doi: 10.3390/ijerph13050493 27187432 PMC4881118

[115] Huang L, Li J, He RY, et al. Quantitative analysis of health risk perception, exposure levels, and willingness to pay/accept of PM2.5 during the 2014 Nanjing Youth Olympic Games. Environ Sci Technol. 2018b;52:13824–13833. doi: 10.1021/acs.est.8b01634 30351043

[116] Zielonka TM. The awareness of pulmonologists and patients with respiratory diseases about the impact of air pollution on health in Poland. J Clin Med. 2021;10:2606. doi: 10.3390/jcm10122606 34204758 PMC8231647

[117] Williams ID, Bird A. Public perceptions of air quality and quality of life in urban and suburban areas of London. J Environ Monit. 2003;5:253–259. doi: 10.1039/b209473h 12729264

[118] Pantavou K, Lykoudis S, Psiloglou B, et al. Air quality perception of pedestrians in an urban outdoor Mediterranean environment: A field survey approach. Sci Total Environ. 2017;574:663–670. doi: 10.1016/j.scitotenv.2016.09.090 27662493

[119] Yang ZJ, Rickard LN, Harrison TM, et al. Applying the risk information seeking and processing model to examine support for climate change mitigation policy. Sci Commun. 2014;36:296–324. doi: 10.1177/1075547014525350

[120] Katsuya T. Public response to the Tokai nuclear accident. Risk Anal. 2001;21:1039–1046. doi: 10.1111/0272-4332.216172 11824679

[121] Huang L, Zhou Y, Han Y, et al. Effect of the Fukushima nuclear accident on the risk perception of residents near a nuclear power plant in China. Proc Natl Acad Sci USA. 2013;110:19742–19747. doi: 10.1073/pnas.1313825110 24248341 PMC3856800

[122] Varni JW, Seid M, Rode CA, et al. The PedsQL: measurement model for the pediatric quality of life inventory. Med care. 1999;37:126–139. doi: 10.1097/00005650-199902000-00003 10024117

[123] Dou H, Zhao Y, Chen Y, et al. Development and testing of the reliability and validity of the adolescent haze related knowledge awareness assessment scale (AHRKAAS). BMC Public Health. 2018;18:734. doi: 10.1186/s12889-018-5638-8 29898700 PMC6000920

