# Supplementary figures and images for: The perception of air pollution and its health risk: a scoping review of measures and methods
Source: Glob Health Action. 2024 Jun 28;17(1):2370100. doi: 10.1080/16549716.2024.2370100 (PMC11216274; doi:10.1080/16549716.2024.2370100)

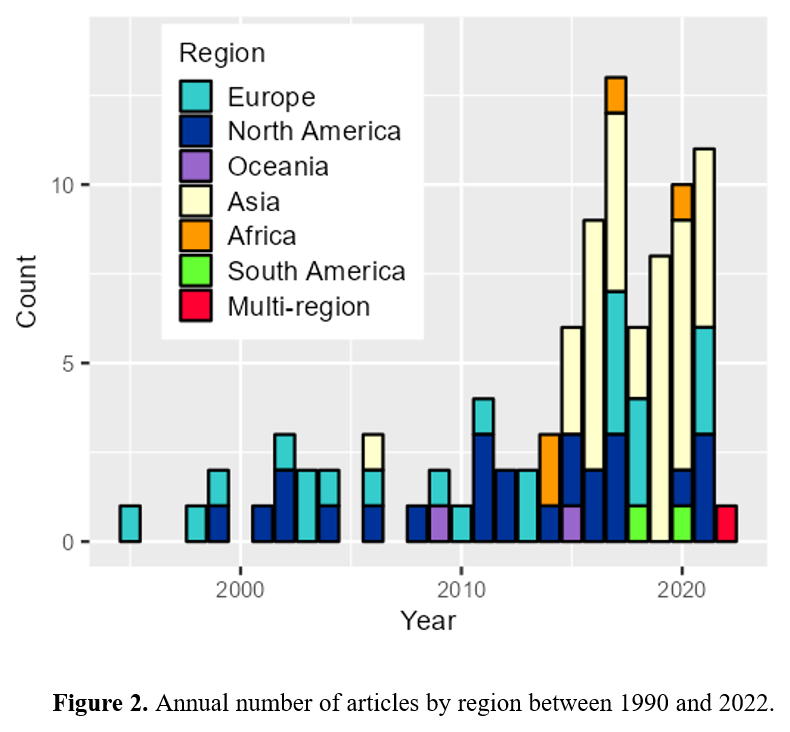

Supplement: Figure2.tif [file ZGHA_A_2370100_SM7964.tif]
